# Supplementary material for: 1,25-Dihydroxyvitamin D3 Provides Benefits in Vitiligo Based on Modulation of CD8+ T Cell Glycolysis and Function
Source: Nutrients. 2023 Nov 6;15(21):4697. doi: 10.3390/nu15214697 (PMC10650610; doi:10.3390/nu15214697)
Supplement: Supplementary file 1 [file nutrients-15-04697-s001.zip › nutrients-2659828-supplementary.pdf]

nutrients

Supplementary Materials

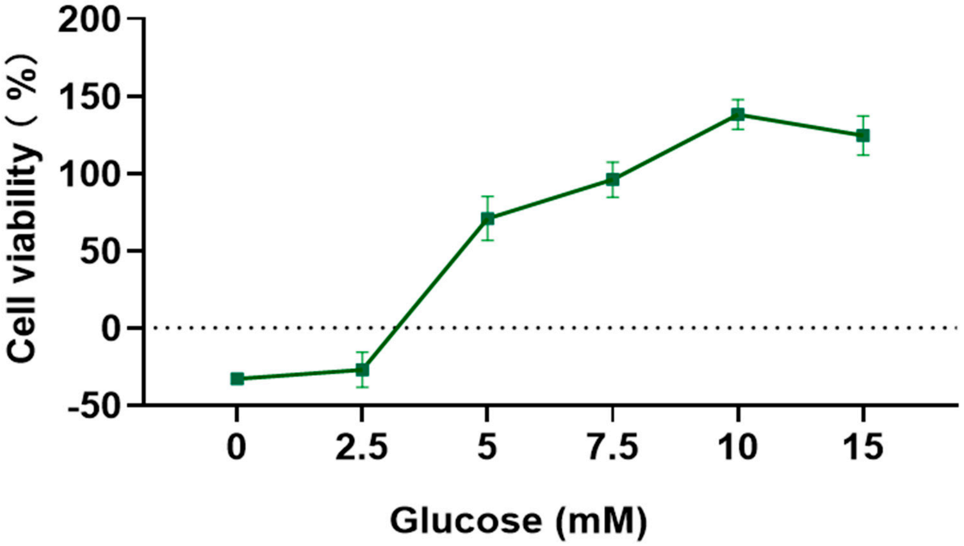

Figure S1. Cell viability of Tall-104 cells in different glucose concentrations cultured for 72 h, determined using CCK-8 analysis.

Table S1. Significantly different genes in peripheral blood mononuclear cell samples from participants receiving 1,25$(OH)_2D_3$ and controls.

Gene name	*p* Value	Log_2FC	Change
ZNF493	0.000514369	−2.847003948	down
SPOP	0.000578038	−2.691764563	down
MID1IP1	0.000702233	−2.386975273	down
RPL11	0.000927457	17.33390635	up
TRAPPC1	0.000931115	−2.112686645	down
MAP1LC3A	0.001107775	−2.752128316	down
TUBA4A	0.001477045	−5.312375851	down
RPS28	0.001580771	−10.11611965	down
CD37	0.001656278	−3.979420153	down
SNRNP70	0.001715045	−3.180022248	down
NDE1	0.001813882	−4.966306472	down
BICD2	0.001848346	−2.28316845	down
CDAN1	0.002203658	−10.47737987	down
EGLN2	0.002295192	−4.870214396	down
DUSP22	0.002315663	−2.131845984	down
IL18	0.002335676	−9.259413617	down
CLINT1	0.00242058	2.433742447	up
RHOU	0.002445738	2.299298122	up
PAK2	0.002649921	−2.120541808	down
MAP4K2	0.002917921	−2.577165752	down
DPEP2	0.003014088	−3.775971003	down
BTF3	0.003174102	6.647639338	up
ARHGEF3	0.003434715	3.874514041	up

S1PR4	0.003460417	−2.859850595	down
ZNF486	0.003465097	−14.51052417	down
TOM1	0.003483513	−3.007592253	down
PFDN5	0.003588983	9.826718666	up
ASXL2	0.003920204	−2.239287816	down
CPPED1	0.004120869	−2.51374911	down
TMX4	0.004465498	−2.038413717	down
IFI44	0.004468412	5.199037022	up
MCM8	0.004558078	−10.24514101	down
PDE4C	0.004857214	−6.808602199	down
DMC1	0.004944945	−14.02246088	down
UBXN4	0.005429135	2.886995483	up
DOCK8	0.005580443	3.166057321	up
SON	0.005721416	2.961136813	up
PANX2	0.005891666	−3.191828602	down
FKTN	0.00610848	−8.213856489	down
FCGR3A	0.00626856	−3.983069812	down
GALK1	0.006432826	−2.504318451	down
SH2B3	0.00682418	2.147315407	up
EIF1	0.007036127	4.122852152	up
PPP4C	0.0071493	−2.186519556	down
LRCH4	0.00715465	−2.531176505	down
SLC44A4	0.007607274	−8.540190884	down
DCAF6	0.007652924	3.930443053	up
PRKY	0.007672196	2.021562744	up
EFR3A	0.007714641	3.0855519	up
UBE2D3	0.007842766	4.713846655	up
AIRE	0.008140488	−10.62758784	down
PCMTD1	0.008214796	3.472529254	up
DAPP1	0.008310967	−10.36001228	down
OCIAD1	0.008862567	−11.74023951	down
ZNF430	0.008885496	−8.701117738	down
HIGD1A	0.008987185	2.285060208	up
SAMD9	0.009048188	2.970010631	up
BIN1	0.009258608	−2.883696545	down
VAV3	0.009538507	3.001688333	up
RELL1	0.009892822	−3.140407112	down
HBB	0.009921654	−4.485940109	down
DNAJA4	0.01015338	2.18075703	up
SRP14	0.010396603	3.65903234	up
LEMD3	0.010616041	2.454027814	up
YIPF3	0.010780933	−2.672600416	down
GLRX	0.010789529	5.464505284	up
GIMAP4	0.011160228	5.790432998	up
THOC7	0.011304209	2.283658253	up
CX3CR1	0.011707332	6.053255623	up
DUXAP3	0.011793122	−13.04794083	down
CHMP5	0.011807181	4.116988545	up
NBPF8	0.011995922	−4.66680721	down
MIF	0.012166957	−2.983745554	down

YBX1	0.012379299	7.281871457	up
VHL	0.012415268	−2.195611652	down
ANKRD12	0.012535208	2.236581891	up
PLIN5	0.012566215	−5.267740959	down
LOC729086	0.012688584	−3.38767488	down
ACP1	0.012900356	2.83881093	up
XPNPEP3	0.012978855	−6.673035916	down
MYADM	0.013002128	−4.424225183	down
ABTB1	0.013107445	−4.268135944	down
RPS24	0.013168479	14.30196138	up
PSMD12	0.013338951	−7.270633059	down
APOBEC3A	0.01355369	−2.478931381	down
RAB11A	0.013815134	2.327765458	up
COPE	0.013903205	−2.157009747	down
ZFAND5	0.014164853	5.456199045	up
PSMA4	0.014165935	4.306809597	up
RPL41	0.014386505	18.48492976	up
LPAR2	0.014640422	−2.144470372	down
CDKN2AIPNL	0.01498333	−12.69944057	down
ARPC4	0.015174996	−3.181261471	down
ABHD5	0.015227893	−2.557281368	down
PDCD10	0.015325921	3.115975322	up
HBG2	0.016003495	33.40663814	up
TST	0.016005571	−2.928785268	down
PRNP	0.016072929	3.533774012	up
TPI1	0.016445236	−2.366161816	down
LOC399900	0.016540279	−10.44139089	down
RNASEK	0.016633924	−3.834313291	down
OAS2	0.016643418	4.502838479	up
PNRC2	0.016661589	3.271739771	up
SESN3	0.016722497	4.329816016	up
DCP2	0.016952567	3.099322111	up
TRIM22	0.016955847	2.991014026	up
PPP1R15A	0.016970743	−2.540486383	down
CHPT1	0.017186307	4.840124293	up
STAT1	0.017448966	4.585159964	up
HBG1	0.017493897	31.28977235	up
CALM3	0.017520142	−3.636926926	down
MKNK2	0.017622919	−4.161188249	down
LY96	0.017703541	5.091224758	up
IL10	0.017865749	−12.10476322	down
VPS37C	0.018513898	−2.562283922	down
SNRK	0.018689567	2.253683142	up
TAGLN2	0.018934981	−2.585136782	down
RAB10	0.019317952	3.78876991	up
AMFR	0.019362202	2.061769961	up
RPLP1	0.019512209	−9.718744679	down
COX7B	0.019698225	2.481819442	up
S100A8	0.019951433	18.52070587	up
CNIH4	0.019994456	3.100313833	up

AURKAIP1	0.020053843	−2.233657563	down
CD52	0.020099214	7.461381203	up
FCGR3B	0.020251361	−7.932913922	down
CYP4F3	0.021176117	−2.379307531	down
CREG1	0.021837992	2.862620488	up
ZBTB33	0.022384181	2.004336355	up
NDUFB3	0.022643895	3.270027827	up
DDX17	0.022804584	−2.085443911	down
ITGB1	0.023204974	5.021816174	up
ZNF302	0.023401206	2.192614297	up
BNIP2	0.023727147	2.169807766	up
RPS4Y1	0.023839606	17.26368493	up
MAGT1	0.024152026	−8.924097264	down
OXR1	0.025334506	2.312969406	up
RPS27L	0.025418053	3.047805588	up
SUMO2	0.025770289	−3.018622699	down
C2orf69	0.025993417	−10.77752958	down
TIMP1	0.026031594	−6.505965799	down
VASP	0.026038931	−2.931645215	down
RPL37A	0.026340798	−6.544284489	down
RPL17	0.026528518	13.27305914	up
FECH	0.026989462	4.404752758	up
CFLAR	0.027371381	−5.986486437	down
PTPRE	0.027504709	2.10973939	up
DOCK10	0.027555289	2.188021125	up
REEP5	0.027671756	3.258784662	up
SACM1L	0.028097594	2.07955278	up
CXXC5	0.028272743	−2.760487161	down
KAT2B	0.028411106	3.801927373	up
TUBA1A	0.028603775	−3.246343481	down
AKR1D1	0.028900515	−7.52828431	down
COX6C	0.028917164	3.959619882	up
GPSM3	0.029039404	−3.900090195	down
ZYG11B	0.029315388	−2.269124011	down
PLAC8	0.029942775	2.891651469	up
NIN	0.029976815	2.298480814	up
COX7C	0.030237873	5.808368838	up
RPL21	0.030496145	8.92081392	up
HSP90AA1	0.030628941	3.476156222	up
DEFA1	0.030673762	24.34192911	up
TMX1	0.031035185	2.994825371	up
TPT1	0.031310834	13.10266041	up
LTB	0.0319833	−5.306168425	down
RPL7L1	0.032526104	−8.648114665	down
SRP9	0.033147126	5.28173456	up
CREB5	0.033174401	−2.413769296	down
RNASE6	0.03339557	−2.340344619	down
ERGIC1	0.034257494	−2.01142471	down
PTMA	0.034272029	−4.331868726	down
DTWD2	0.034830397	−10.2274216	down

RPS17	0.035226206	13.64381847	up
SNX3	0.035328306	4.912366927	up
TUBA1C	0.035471962	−2.129728252	down
FOXN2	0.035573563	2.515327543	up
RPL31	0.035833984	11.50873551	up
RPL35A	0.035941589	8.316620848	up
ATP6V0E1	0.035955778	−3.201300061	down
PJA2	0.03670776	4.739779747	up
RSL24D1	0.037044387	4.938108358	up
AKT1	0.037503369	−2.247791605	down
SIPA1	0.037877436	−2.156557009	down
AHR	0.037964929	−11.15076124	down
FKBP14	0.038143705	−10.32910884	down
CCM2	0.038446456	−2.312660243	down
CPVL	0.038717794	3.842546173	up
SMCR5	0.038792817	−2.758201852	down
TNS1	0.038944471	3.062504835	up
PSMC6	0.039068213	2.341921491	up
UBC	0.039918996	−4.695739979	down
CGGBP1	0.040071979	2.708629643	up
IL17RD	0.040092731	−10.49800987	down
ATP6V0B	0.040528364	−3.119937176	down
ZNF669	0.040534514	−3.175903981	down
TMEM181	0.040744268	2.197310897	up
CLEC4A	0.040960614	2.185286265	up
PDCD4	0.041029048	−7.198580811	down
NDUFS8	0.041202754	−2.072312508	down
YRDC	0.041437084	−9.857209852	down
CSF2RA	0.041671856	−7.882229621	down
RSAD2	0.042138575	3.651948084	up
TSC22D3	0.042174533	−4.707836886	down
XBP1	0.042442808	2.917357778	up
MFSD11	0.042746098	−3.613888319	down
ZNF738	0.042874804	−10.70047248	down
TRABD	0.042946168	−2.275995511	down
LOC100133177	0.043174457	−5.714268039	down
SNRPG	0.043369287	3.75063538	up
DOCK11	0.043906928	2.472411906	up
SPTLC1	0.043940578	−9.843500377	down
RPL6	0.043994871	9.609928794	up
SP110	0.044237893	−2.540211282	down
BLZF1	0.044306785	−11.68481785	down
PNPT1	0.044323959	−11.34073405	down
TMEM123	0.044796971	4.541145669	up
MBNL1	0.044863903	3.795921965	up
JMJD1C	0.04489485	2.478178889	up
TNFRSF14	0.044931191	−2.122607388	down
TXN	0.045072016	3.646943035	up
RPL30	0.045187766	9.245638309	up
TAX1BP1	0.045214698	3.061151707	up

F11R	0.045297632	−2.322989037	down
RWDD1	0.045422849	2.830711154	up
XIST	0.04555491	−3.404287145	down
SRRD	0.045619599	2.042074231	up
TOMM7	0.046213016	7.291135907	up
LSM1	0.046381017	2.521023601	up
RPL36AL	0.046808592	3.941950674	up
FAM104A	0.046972549	5.980691838	up
IFIT2	0.047031902	7.616260826	up
MS4A7	0.04730251	2.439489777	up
RYBP	0.047741859	−2.220611485	down
ZNF14	0.047910039	−11.99031792	down
FCGRT	0.048016549	−3.23669666	down
TMEM17	0.048035512	−8.742970026	down
USP10	0.048252388	−2.859232616	down
LIPA	0.048359305	2.876972112	up
ACRBP	0.04845519	−2.062230895	down
SLC38A2	0.048503624	2.210704313	up
BIRC2	0.048856428	2.710496109	up
VPS41	0.048999342	−5.68558612	down
DNAJA1	0.049852133	2.914161501	up

Total 244 aberrant expressed genes were identified, including 117 up- and 127 down-regulated genes. $p < 0.05$, FDR < 0.05 and $|Log2FC| > 2$.
